# Experiences of mental healthcare users and their families when interacting with SAPS: A qualitative study

**DOI:** 10.4102/sajpsychiatry.v31i0.2435

**Published:** 2025-06-11

**Authors:** Vuyokazi Kabane, Yumna Minty, Barry L. Viljoen

**Affiliations:** 1 Department of Psychiatry, Faculty of Health Sciences, University of the Witwatersrand, Johannesburg, South Africa; 2 Department of Psychiatry, Division of Clinical Psychology, Faculty of Health Sciences, University of the Witwatersrand, Johannesburg, South Africa

**Keywords:** mental healthcare users, South African Police Services, qualitative study, phenomenology, mental health crises, stigma, criminalisation, emergency response

## Abstract

**Background:**

In South Africa, the South African Police Service (SAPS) is often called to facilitate access to mental healthcare for individuals with severe behavioural disturbances posing risks to themselves or others, as outlined in the Mental Health Care Act (2002). Understanding the experiences of mental healthcare users (MHCUs) and their families during these encounters is essential for improving mental health services.

**Aim:**

To explore the experiences of MHCUs and their families during interactions with SAPS when seeking assistance for hospital admission, focusing on perceptions of SAPS attitudes and responses.

**Setting:**

The study was conducted at the outpatient and inpatient psychiatric departments of Chris Hani Baragwanath Academic Hospital in Southern Johannesburg.

**Methods:**

Between June and December 2023, 15 semi structured interviews were conducted with five inpatients, five outpatients, and five family members. Interviews were audio-recorded, transcribed verbatim, and analysed inductively to identify emergent themes.

**Results:**

Five major themes emerged, highlighting confusion, fear, and feelings of criminalisation. Participants reported inadequate SAPS support, with the escalation of force often worsening anxiety and distress. Families were frequently misinformed, including being advised to call an ambulance, which delayed care. SAPS involvement often led to public embarrassment, affecting MHCUs’ dignity.

**Conclusion:**

SAPS responses to mental health emergencies frequently increased distress among MHCUs and families. The findings highlight the need for SAPS training and clear protocols to manage psychiatric crises with care and sensitivity.

**Contribution:**

This study informs interventions aimed at improving SAPS–MHCU interactions.

## Introduction

Mental healthcare users (MHCUs) and their families frequently interact with the South African Police Service (SAPS) during acute mental health crises and when hospital admission may be necessary. Acutely ill individuals often refuse hospitalisation or medication and may behave in a manner that is perceived as threatening, prompting families to seek SAPS intervention for safe transportation to medical facilities. *Despite the Mental Health Care Act (No. 17 of 2002)*^1^ outlining the role and responsibility of police officers in such situations, the experiences of MHCUs and their families and their perceptions of the assistance received during these interactions are not well documented.

Stigma and discrimination against MHCUs from various sources are well studied, highlighting the dual challenge of managing symptoms and combating societal misconceptions.^2,3^ These negative attitudes can come from various avenues, including civil staff (such as police officers). These challenges may lead to social exclusion and adverse health outcomes.^3,4^ Studies in South Africa, such as those by Egbe et al.,^4^ and Booysen et al.,^4^ underscore the severe impact of stigma on MHCUs, contributing to their marginalisation and impeding access to community activities, healthcare, work opportunities and education.

As defined by Section 40 of the *Mental Health Care Act (MHCA) No. 17 of 2002*, the SAPS’s role aims to prevent the unlawful arrest and detention of MHCUs and ensure that they receive appropriate care in health establishments.^1^ Specifically, the Act mandates that SAPS members must facilitate the safe transportation of mentally ill individuals – who may pose a risk to themselves or others – to designated mental health establishments, thereby ensuring these individuals receive the necessary care rather than being wrongfully detained.^1^ However, practical implementation faces challenges. Jonsson et al.^5^ highlighted the lack of preparedness and training among SAPS members when dealing with MHCUs, while Mjali’s study revealed that many SAPS members were unaware of Section 40 of the Act, leading to potential mishandling of MHCUs.^6^

Studies indicate that MHCUs often experience interactions with SAPS as stigmatising and criminalising, exacerbating their distress.^3,4,5,6^ Although not a South African study, Watson et al., emphasised the need for more research on MHCUs’ perspectives to inform training and improve police responses. These findings are relevant in South Africa, where similar challenges in law enforcement’s interaction with MHCUs have been reported, highlighting a significant gap in training and appropriate response protocols.^3^ Given these challenges, this study seeks to address the research question: What is the lived experience of MHCUs and their families regarding the service rendered by SAPS when interacting in an acute emergency setting to access healthcare and treatment? To explore this, the study aimed to address these gaps in information by providing a qualitative phenomenological description of the lived experiences of MHCUs and their families when interacting with SAPS.

## Research methods and design

### Study setting

The study was conducted at Chris Hani Baragwanath Academic Hospital (CHBAH) in Soweto, Johannesburg, South Africa. The CHBAH, one of Africa’s largest hospitals, provides specialised psychiatric services through its outpatient department and inpatient wards. The Department of Psychiatry treats conditions such as schizophrenia, bipolar disorder, major depressive disorder and substance-induced psychotic disorder.^7,8^ The psychiatric unit has four adult wards with 155 beds, maintaining a 90% occupancy rate, and a new 21-bed Psychiatric Admission Ward was added in 2023.^7,8^ The CHBAH was chosen for its high patient volume, referral role and frequent SAPS involvement in psychiatric emergencies. Participants included psychiatric inpatients, outpatients and their family members.

### Study design

This study employed a qualitative descriptive phenomenological approach to systematically describe the experiences of MHCUs and their family members during their interactions with SAPS.^7,8,9^ Interview procedures and transcription preparation followed established best practices in qualitative interviewing and data handling.^10,11^

### Study population and sampling strategy

The study population comprised two distinct groups: (1) mental healthcare users (MHCUs), including inpatients and outpatients and (2) family members who interacted with SAPS during a mental health crisis. These groups were analysed separately to explore their unique perspectives and experiences. Recruitment was carried out through a combination of purposive and convenient sampling. Participants were selected based on their attendance at CHBAH, making them readily accessible to the researcher. Purposive sampling was used to identify participants who had previously interacted with SAPS during an admission to the hospital.^9^ The MHCUs were assessed for their ability to provide informed consent by their treating doctors in the outpatient department and inpatient wards. Psychotic patients were included, provided their condition did not impair their ability to engage meaningfully with the study. Those who were disorganised or had significant thought disorder that affected comprehension and participation were excluded.

A sample size of 15 participants was determined to be adequate for the study, following recommendations by Leedy and Ormrod.^9^ This included 10 MHCUs (divided equally between inpatients and outpatients) and five family members (Table 1).

**TABLE 1:** Characteristics of sample population.

Participant number	Category	Age (years)	Gender	Level of education	Work status
P1	Inpatient	43	Male	Degree in accounting science	Employed
P2	Inpatient	28	Male	Grade 11	Unemployed
P3	Inpatient	40	Male	Grade 12	Self-employed
P4	Family member	50	Male	Grade 11	Unemployed
P5	Outpatient	56	Male	Grade 12	Disability Grant
P6	Family member	63	Female	Grade 12	Pensioner
P7	Outpatient	49	Female	Grade 9	Disability Grant
P8	Outpatient	53	Male	Grade 12	Disability Grant
P9	Inpatient	32	Male	Grade 10	Employed
P10	Outpatient	34	Male	Grade 11	Disability Grant
P11	Family member	47	Female	Degree in social science	Employed
P12	Outpatient	21	Male	Grade 12	Unemployed
P13	Inpatient	33	Male	Grade 12	Unemployed
P14	Family member	68	Male	Grade 12	Pensioner
P15	Family member	56	Female	Degree in social work	Employed

### Data collection procedure

Data were collected through semistructured interviews and recorded using the OTTER application, which transcribes audio recordings into text. The interviews for outpatients and inpatients were conducted in the outpatient department and in the wards, respectively. Family members were interviewed in the outpatient department or during visiting hours in the wards. The interviews took place between 21 June 2023, and 19 October 2023, with durations ranging from 12 to 28 min. Interview questions focused on the participants’ interactions with SAPS, how they responded to these interactions, and their feelings about them. For many participants, recalling their experiences was distressing, as they vividly remembered the events. The MHCUs often described feelings of fear and confusion, while family members frequently expressed frustration with police responses and delays. Examples of interview questions include:

- Tell me about the time you had to contact or your family had to contact the SAPS?
- Tell me about the response of SAPS?
- What information were you given by the members of SAPS?
- How do you feel about the interaction with members of SAPS?
- Would you call SAPS again for their assistance?

All 15 interviews were audio recorded and converted to transcripts using the OTTER application. The transcripts were then translated verbatim with the aid of the recordings. The interviews were conducted mostly in English but some participants used Zulu, depending on their preference. These transcripts were translated into English by the researcher. The transcriptions were reviewed multiple times, with spelling and grammatical errors corrected and disfluencies removed to ensure accuracy.^12,13,14,15^ Field notes were also taken to provide additional context.

### Data analysis

Data analysis followed the principles of descriptive phenomenology, employing an inductive, line-by-line coding approach.^16,17,18,19,20^ The process began with data familiarisation, which involved thoroughly reading the transcripts to gain an in-depth understanding of the participants’ experiences.^14,15^ Initial codes were generated from the raw data and organised into categories, which were then refined into broader themes.^13^ Bracketing was applied throughout the analysis to minimise researcher bias, with the researcher engaging in reflexive journaling and regular debriefing sessions with Authors 2 and 3 to ensure that themes emerged from the data rather than preconceived assumptions This iterative process ensured an accurate and comprehensive representation of participants’ experiences, and the formation of themes is illustrated in Table 2. An independent code was not used to verify the themes. Instead, themes were developed inductively from the data, with oversight from Author 2 and Author 3. Regular discussions were held to refine themes, ensure consistency and achieve consensus.

**TABLE 2:** Inductive framework illustrating the development of themes from code categories.

Code categorisation	Themes
• Criminalisation	1. The presence of South African police service, physical force and criminalisation
• Feelings towards the SAPS	2. Inadequate support from SAPS for mental health emergencies
• Assistance provided •Manpower •MHCU behaviour	3. Escalation of force due to lack of assistance
• Physical force by SAPS •Attitudes by SAPS	4. Physical force used by SAPS and attitudes towards mentally unwell individuals
• Reputation, desires and communication	5. Rebuilding self-identity: navigating public perception and personal dignity, returning to community

SAPS, South African police service.

### Trustworthiness

Trustworthiness was ensured through credibility, dependability, confirmability and transferability. Triangulation was achieved by incorporating perspectives from both MHCUs and family members, strengthening credibility. Prolonged engagement and detailed documentation further enhanced the accuracy of findings. Dependability was ensured through a structured interview guide and regular discussions among the research team. Confirmability was maintained via an audit trail, including verbatim transcripts and systematic coding documentation. Transferability was supported by detailed descriptions of the study setting and participant demographics, ensuring applicability to similar contexts.

### Ethical considerations

Ethical clearance to conduct this study was obtained from the University of the Witwatersrand Human Research Ethics Committee (HREC) (No. M220919). All participants provided informed, written consent, with confidentiality maintained throughout the study. For inpatients, capacity to provide consent was carefully evaluated and confirmed by their treating clinician.

Informed consent was obtained from all participants. Treating doctors assessed MHCUs’ capacity to consent and conducted mental state examinations to determine suitability. Those with thought disorder or disorganisation affecting comprehension were excluded. Confidentiality was maintained through unique identifiers, and access to data was restricted to the research team.

Interviews were conducted in private settings to minimise distress and participants could withdraw at any stage without consequence. Psychological support was available, with referrals to treating clinicians if needed.

## Results

The study identified five themes and one subtheme that encompass the experiences of MHCUs and their families during their interactions with SAPS. These themes are described in more details next:

### The presence of South African police service, physical force and criminalisation

Participants reported feeling confused and fearful because of the abrupt and forceful presence of SAPS. They also reported being made to feel like criminals because of the actions and attitudes of officers.

Participant 13 described:

‘They just tell me to get inside the van, “You didn’t act properly to your parents, you must get inside the holding cell, will take you out of the holding cell and take you to hospital and then the doctors will take over work for me”’. (P13, Inpatient, 33, Male, Grade 12)

Another participant (participant 3) echoed:

‘But this second time it was like I felt more like a criminal, like someone who’s done a huge crime or something’. (P3, Inpatient, 40, Male, Grade 12)

Participant 1 shared:

‘The restraining that is roughly done. You know, it’s not the, like this, it feels like you’re a hardened criminal. You just murdered someone, you know, like, you were very aggressive on the street or something’. (P1, Inpatient, 43, Male, Degree in accounting science)

Participant 2 shared:

‘I feel like a criminal now because now we were fighting. You know I feel like a criminal. “Why bengasho kahle ukhuti ye mfethu uya esibedlele relaxer?”, (“Why don’t they tell me politely that I am being taken to hospital and I should relax?”), I feel like a criminal woza ndoda’. (P2, Inpatient, 28, Male, Grade 11)

### Inadequate support from South African police service for mental health emergencies

Families often struggled to get timely and effective assistance from SAPS. They reported that they may be instructed to call an ambulance first or get a referral letter, delaying necessary help.

Participant 6 recounted:

‘They say no, they can’t they have to wait for the ambulance. But I told him that before police helped me. They put him into the van and bring him to Bara. Why can’t you do it. No, it’s a rule now. We can’t take him now. You have to wait for the ambulance, the ambulance didn’t come’. (P6, Family member, 63, Female, Grade 12)

Participant 15 recalls:

‘The first time I went there a few times personally. And they tell you straight up it’s not our problem, call the ambulance. And then you can’t just call the ambulance they want to know if it’s really serious or so on. So if you know, that’s the experience I had with the police’. (P15, Family member, 56, Female, Degree in social work)

According to them:

‘We got to go back to the hospital and get a letter from them to pick him up and you know what? A problem that is, the incident is occurring now so we don’t have time to go back get a referral letter.’

Participant 4 states:

‘At first we did call the cops. They came and we took them to the hospital. And then for the second time and the third time, I went straight to the police station and asked for assistance. At some point if you call them, they give you a reference number then you wait up until the sun goes down’. (P4, Family member, 50, Male, Grade 11)

### Escalation of force because of lack of assistance

Families often found themselves managing mentally unwell individuals alone because SAPS either refused to assist when they did arrive, prioritised other emergencies or insisted that families first obtain referral letters from healthcare professionals. Instead of providing immediate support, SAPS would inform families to contact emergency medical services (EMS) to handle the situation. These barriers left families vulnerable, forced to deal with potentially hazardous situations without the necessary training or assistance, increasing the risk of escalation.

Participant 4 described:

‘It becomes difficult because you are calling this side and that side. Even that ambulance when it arrives it tells you, the person must be escorted by the police, you see. Because I saw last time when I had to bring my uncle, he was very violent. He broke windows, doing a lot of things. So, I ended up noticing that we were not managing. I ended up having to tying his hands and feet with a rope. So that at least we can sleep’. (P4, Family member, 50, Male, Grade 11)

Participant 11 explained:

‘They came and they spoke to her and then they left. They said they were rushing somewhere; they had a call. We tried to get numbers; we saw the situation was getting worse until I decided to get manpower and they tied her up’. (P11, Family member, 47, Female, Degree in social science)

Participant 14 reported that he no longer calls the police:

‘No, I don’t. I get two or three or four guys together to calm him down. And he comes by force; we take him to the hospital’. (P14, Family member, 68, Male, Grade 12)

### Physical force used by South African police service and attitudes towards mentally unwell individuals

Participants recounted instances of rough handling, dismissive language and forceful driving by SAPS, which often exacerbated their distress. For example, Participant 1 emphasised that not all mentally ill individuals are violent and should be assessed individually. Additionally, derogatory attitudes and minimal interaction from SAPS contributed to the stigma faced by MHCUs and their families.

Participant 1 noted:

‘I’m not a violent person, and I think police should assess how much force they need to apply because we’re not all the same. I’m not a violent person. I can comply. Yes, I’ll be in a state, but I can comply. I can comply and get into the ambulance’. (P1, Inpatient, 43, Male, Degree in accounting science)

Participant 7 stated:

‘Anything they do. They’re rough. The way they tie me up. Some will tie you up, some will pull you. The way they drive also, their van will sway’. (P7, Outpatient, 49, Female, Grade 9)

### Rebuilding self-identity: Navigating public perception and personal dignity

In the aftermath of mental health crises, individuals faced significant challenges in rebuilding their self-identity and coping with public perception. Participants preferred ambulance escorts over police, noting the respectful and caring approach of ambulance personnel.

Participant 8 appreciated:

‘I like the ambulance because when they arrive, they check me and ask me questions … When the police arrive, they do not check me; they only see the side that I am sick … So the ambulance is better, and they are able to ask questions’. (P8, Outpatient, 53, Male, Grade 12)

Recovering personal identity and dealing with embarrassment after an interaction with SAPS was witnessed by the community was also reported to be a concern for participants.

Participant 9 stated:

‘I feel like I don’t know where to look when I walk in the street … I just force myself and go out to face society because the whole community knows me as a respectable person … But I keep my head up high, break my fears, and tell myself everything happened as it was meant to. I am not going to let people let me down. I am not a coward’. (P9, Inpatient, 32, Male, Grade 10)

## Discussion

Booysen et al.^3^ reported that many MHCUs in South Africa face substantial barriers to accessing support, which further exacerbates their vulnerability during interactions with SAPS. The National Mental Health Policy Framework and Strategic Plan (2013–2020)^21^ also highlights the gaps in support systems for MHCUs and the need for more integrated care pathways, which currently remain insufficiently addressed.

A recurring theme in this study was the sense of criminalisation experienced by MHCUs during interactions with SAPS, where individuals were often treated as criminals rather than people in crisis needing medical assistance.^5,22^ Jonsson et al.^5^ found that SAPS officers frequently lack the training required to appropriately distinguish between criminal behaviour and acute mental health symptoms, leading to significant misinterpretations. These misinterpretations arise because mental illness may manifest itself in ways that can resemble criminal conduct, such as erratic behaviour, aggression or noncompliance.^22,23^ Without sufficient understanding of mental health and its signs and symptoms, police officers tend to perceive these actions as wilful disobedience or threats to public safety, thus responding with force or arrests instead of deescalation or medical intervention.^5,22^

Mnguni et al.^22^ emphasised that the criminalisation of MHCUs is a systemic issue, wherein police officers are focused on maintaining control and containment rather than recognising the need for medical care. These responses reflect broader societal stigma towards mental illness, as noticed by Booysen et al.,^3^ which is perpetuated by law enforcement practices that treat mental health crises as public order problems rather than emergencies requiring specialised care.

Participant accounts in this study reinforced these findings. Such interactions align with concerns raised by Lamb et al.,^23^ who observed that police interactions with mentally ill individuals often lack appropriate mental health crisis intervention training. As a result, the recurrence of this theme in this study highlights the urgent need for improved police training and reform in how mental health crises are approached, as the continued criminalisation of MHCUs deepens their marginalisation and exacerbates their distress.^5,22^

The *Mental Health Care Act (2002)* mandates that SAPS are involved in transporting MHCUs to healthcare facilities, but without proper training, these interventions frequently result in further marginalisation and stigmatisation.^1^ This issue is mirrored globally, where inappropriate police involvement has been shown to exacerbate crises, leading to detrimental outcomes for those involved.^23^ *The Al Jazeera*^24^ report on the tragic death of a disabled teenager after a police shooting in South Africa starkly illustrates the severe consequences of inadequate training and inappropriate law enforcement intervention during a mental health crisis.

Participants consistently reported inadequate support from SAPS during mental health emergencies, often being told to wait for ambulances, which led to delays in treatment and increased distress for MHCUs and family members. This inconsistency in response is a reflection of broader systemic challenges within the South African mental healthcare system.^2,3^ The *Mail & Guardian*^25^ highlighted similar issues, describing the challenges faced in accessing timely psychiatric care because of the disjointed coordination between components of emergency services. Green^26^ also emphasised that police, when acting as frontline mental health workers, often lack the necessary training and support systems to effectively manage mental health crises, which reflects a lack of structural integration. Mjali^6^ found that inadequate support is particularly prevalent in rural South Africa, where systemic barriers such as a lack of specialised mental health training for SAPS officers and limited access to mental health services lead to significant delays in crisis intervention. Geographic isolation and bureaucratic requirements, such as referral letters, further exacerbate these challenges, leaving families to manage crises for extended periods.^6^

The lack of assistance from SAPS often led families to attempt to manage crises on their own, resulting in potentially hazardous situations.^5,6^ The escalation of force by family members or the MHCU’s own behaviour often resulted from the absence of timely law enforcement support, placing both the mentally unwell individual and those around them at risk.^5,6^ Internationally, Teplin^27^ highlighted the importance of effective police intervention in managing mental health emergencies, finding that poorly handled interventions can exacerbate rather than mitigate a crisis.

The excessive physical force used by SAPS, as reported by participants, appears to be driven by a lack of understanding of mental health issues as well as discriminatory attitudes.^5,28^ Jonsson et al.^5^ indicated that these discriminatory attitudes often stem from insufficient training on mental health issues, which leads to inappropriate use of force during crisis situations. Globally, Kane et al.^28^ found similar patterns, where the use of force by law enforcement is often excessive when officers are not adequately trained to manage mental health crises. Jonsson^29^ emphasised the need for ongoing training of SAPS officers on their roles and responsibilities, mental healthcare users’ rights, and general mental health-related issues, highlighting that such training is essential to improve responses during mental health crises. However, much broader implementation of such training is required to create meaningful change in attitudes and responses within law enforcement.

The negative impact on participants’ self-esteem and dignity when being escorted by police to hospital was a significant concern for participants, who expressed a preference for ambulance transportation, which they perceived as more respectful and empathic.^3,30^ This preference is indicative of the stigma associated with law enforcement involvement during such situations, reflecting broader societal biases that view individuals experiencing crises as disruptive rather than in need of care. Booysen et al.^3^ found that police involvement often leads to public embarrassment and damages the community reputation of MHCUs, thereby contributing to further alienation. Such stigma significantly impairs MHCUs’ self-esteem and can hinder their recovery.^2,3^ The South African Federation for Mental Health^30^ has also stressed the need for a human rights-based approach to mental healthcare, highlighting that respectful treatment by all service providers is crucial for preserving the dignity of MHCUs.

The findings of this study point to an urgent need for SAPS to adopt a more informed and compassionate approach when responding to calls for assistance from MHCUs and their families. Mandatory training for law enforcement officers focusing on mental health awareness, deescalation techniques and stigma reduction is essential for improving interactions between SAPS and MHCUs.^3,5,25^ Such initiatives should be expanded beyond pilot programmes and integrated into the core training for SAPS officers. Internationally, the Crisis Intervention Team (CIT) model has proven successful in reducing arrests and improving the outcomes of mental health crises.^31,32,33,34^ Adopting a similar model in South Africa, involving mental health professionals, social workers and specially trained officers, could prevent unnecessary criminalisation of MHCUs, provide more empathic care and ensure that the dignity of MHCUs is upheld. Moreover, it is important to recognise the socio-economic and cultural context within South Africa; implementing CIT or a similar model would require careful adaptation to ensure that these programmes address local challenges such as resource limitations and historical inequalities in healthcare access.^21^

The systemic barriers MHCUs and their families face, particularly the lack of integrated response mechanisms, highlight the need for significant reform.^3,21^ The fragmented coordination between SAPS and healthcare services often leads to delays and increased distress for MHCUs. The National Mental Health Policy Framework and Strategic Plan (2013–2020)^21^ emphasises the importance of integrated care pathways, yet implementation remains inadequate. Developing these pathways is crucial, as they would enhance emergency response coordination and help establish continuity of care, ensuring that MHCUs receive appropriate follow-up after initial interventions. Mjali^6^ found similar barriers in rural areas, where the absence of coordinated care leaves families unsupported and increases the burden on communities to manage crises without adequate resources. International studies, such as those by Teplin,^27^ Dupont and Cochran,^29^ and Kane et al.,^28^ consistently demonstrate that coordinated care pathways improve crisis outcomes, reduce stigma, and foster better community relationships with law enforcement. Establishing clear guidelines and improving collaboration between SAPS and medical services would help create a more consistent and compassionate response to mental health crises, ultimately improving outcomes for MHCUs and their families.

### Strengths and limitation

The qualitative nature of the study allowed for a detailed exploration of the lived experiences of MHCUs and their families during interactions with SAPS, providing rich and compelling data. However, the small sample size of 15 participants limits the generalisability of the findings, although they may be transferable to similar settings. While qualitative research relies on the researcher’s interpretive skills, bracketing was employed to minimise personal biases and ensure that themes emerged from participants’ narratives rather than researcher assumptions. Additionally, the volume of qualitative data collected necessitated extensive time for analysis and interpretation.

## Conclusion

The findings of this study, examined through a phenomenological lens, underscore the urgent need for SAPS to adopt a more compassionate and informed approach in handling mental health crises. The current challenges faced by MHCUs and their families highlight systemic gaps, particularly in the coordination between law enforcement and medical services. These gaps often result in exacerbated distress and can lead to the unnecessary criminalisation of individuals with mental illness.

To address these issues, it is imperative that SAPS improves its coordination with healthcare providers and ensures that police officers receive comprehensive training focused on mental health awareness, deescalation techniques, and the importance of preserving the dignity of MHCUs. Developing and implementing protocols that prioritise the well-being and support of MHCUs is essential for a more humane and effective response to mental health emergencies. Moreover, the study identified a critical issue of misinformation provided by SAPS, specifically the incorrect advice that families must call an ambulance first during a mental health crisis. This advice contradicts the *South African Mental Health Care Act*, which mandates a more direct involvement of law enforcement in such situations.

By addressing these issues, SAPS can significantly improve its response to mental health crises, thereby enhancing the overall well-being of individuals with mental illness and their families. These steps are not only essential for fostering a more compassionate approach to mental health but also for ensuring that the response to such crises is both legally sound and aligned with best practices in mental healthcare.
